# Preparation, characterization, and property evaluation of *Hericium erinaceus* peptide–calcium chelate

**DOI:** 10.3389/fnut.2023.1337407

**Published:** 2024-01-09

**Authors:** Haofeng Gu, Lei Liang, Yongning Kang, Rongmiao Yu, Jiahao Wang, Dan Fan

**Affiliations:** School of Modern Agriculture and Biotechnology, Ankang University, Ankang, China

**Keywords:** *Hericium erinaceus*, peptide-calcium chelate, binding mechanisms, gastrointestinal digestion, calcium bioavailability

## Abstract

Recently, owing to the good calcium bioavailability, peptide–calcium chelates made of various foods have been emerging. *Hericium erinaceus*, an edible fungus, is rich in proteins with a high proportion of calcium-binding amino acids. Thus, mushrooms serve as a good source to prepare peptide–calcium chelates. Herein, the conditions for hydrolyzing *Hericium erinaceus* peptides (HP) with a good calcium-binding rate (CBR) were investigated, followed by the optimization of HP–calcium chelate (HP-Ca) preparation. Furthermore, the structure of the new chelates was characterized along with the evaluation of gastrointestinal stability and calcium absorption. Papain and a hydrolysis time of 2 h were selected for preparing *Hericium erinaceus* peptides, and the conditions (pH 8.5, temperature 55°C, time 40 min, and mass ratio of peptide/CaCl_2_ 4:1) were optimal to prepare HP-Ca. Under this condition, the chelates contained 6.79 ± 0.13% of calcium. The morphology and energy disperse spectroscopy (EDS) analysis showed that HP-Ca was loose and porous, with an obvious calcium element signal. The ultraviolet–visible (UV) absorption and Fourier transform infrared spectroscopy (FT-IR) analysis indicated that calcium possibly chelates to HP via interaction with free -COO- from acidic amino acids and *C* = O from amide. HP-Ca displayed good stability against stimulated gastrointestinal digestion. Moreover, HP-Ca significantly improved the calcium absorption by Caco-2 epithelial cells. Thus, HP-Ca is a promising Ca supplement with high calcium bioavailability.

## 1 Introduction

Calcium is an indispensable element for humans and is directly related to bone and teeth growth, intracellular signal transduction, and hormone secretion (1, 2). However, calcium deficiency is very common in old people, adolescents, and pregnant women (3). Therefore, various types of Ca supplements have been prepared including ionized calcium (calcium gluconate/lactate and calcium carbonate) and amino acid-Ca chelates. However, these supplements have some shortcomings: (1) once ingested, ionized calcium forms insoluble complexes with oxalic acid or phytic acid, easily causing constipation or diarrhea and leading to low calcium bioavailability (4); and (2) the cost of amino acid calcium is much higher than others, and the complex easily leads to adverse reactions (fat oxidation) (5). Recently, cost-effective peptide–calcium supplements have been developed. Owing to the chelation between Ca^2+^ and peptides, once ingested, the peptides protect calcium from forming insoluble complexes in the intestine, thus improving calcium bioavailability (4). Furthermore, the intact peptides can be quickly transported and absorbed by intestinal epithelial cells via pinocytosis or the paracellular pathway (6), and calcium chelated by peptides can be absorbed at the same time. Thus, peptide–calcium supplements display outstanding bioavailability.

In recent years, many food-derived peptides have been used to prepare peptide–calcium supplements, such as peptides from marine animals (7), bone collagen (6, 8), wheat protein (9), and milk (10). These chelates display good calcium bioavailability, confirming that these dietary peptides facilitate calcium absorption. The CBR of peptides is affected by hydrolyzing conditions [type of proteases, hydrolysis time, or hydrolysis degree (HD)] and the origin of proteins. However, the hydrolyzing condition often varies with the origin of proteins (11). Moreover, the CBR of the peptides is also closely related to the preparing condition (temperature, time, pH, and others) of peptide–calcium chelates. Therefore, to prepare new peptide–calcium chelates, not only should the hydrolyzing condition be studied, but the preparing condition of the peptide–calcium complex should also be optimized.

*Hericium erinaceus* is an edible mushroom containing various substances with biological activities, including polysaccharides, sterols, and erinaceolactones (12). The mushroom also contains a high amount of protein, accounting for 26–29% of the dry weight (13). Moreover, glutamate, aspartic acid, lysine, and serine are rich in this mushroom, all of which can provide binding sites to Ca^2+^ (14). Thus, *Hericium erinaceus* is an excellent candidate for the preparation of calcium-binding peptides that has only been sparsely studied.

Therefore, the objective herein was to investigate the optimal hydrolyzing condition (type of proteases and hydrolysis time) of HP with good CBR; secondly, the preparation condition of *Hericium erinaceus* peptide–calcium chelates was optimized by the Box–Behnken design (BBD); thirdly, the characterization of the new chelates was investigated, and the gastrointestinal stability and calcium absorption of the complexes were evaluated. The data obtained facilitate the development of new food-derived peptide–calcium supplements and the improvement of the diversification of functional foods made of *Hericium erinaceus.*

## 2 Materials and methods

### 2.1 Materials and reagents

*Hericium erinaceus* was provided by Guohua Agriculture and Forestry Technology Development Co., LTD (Ankang, China). Fluo-3AM, papain (800 U/mg), neutral protease (100 U/mg), Protamex (120 U/mg), pepsin (3,000 U/mg), alkaline protease (200 U/mg), and trypsin (2,500 U/mg) were bought from Shanghai Biotechnology Co. LTD (Shanghai, China). The Caco-2 cell line was provided by the China Center for Type Culture Collection (Wuhan, China). The penicillin–streptomycin mixture, fetal bovine serum (FBS), CCK8 kit, non-essential amino acid (NEAA) solution, and DMEM medium were bought from Solarbio Co., Ltd. (Beijing, China).

### 2.2 Extraction of *Hericium erinaceus* proteins

The hot air-dried (55°C, 24 h) *Hericium erinaceus* fruit body was smashed into powders and dissolved in distilled water at the ratio of 1:35 (g/mL). The pH of the mixture was adjusted to 10, and *Hericium erinaceus* proteins were extracted at 65°C for 5 h. The mixture was centrifuged (2,000 × *g*, 15 min), then the pH of the supernatant was adjusted to 3.5 to precipitate the proteins for 4 h, followed by centrifugation (2,000 × *g*, 15 min). The precipitated *Hericium erinaceus* proteins were collected and freeze-dried.

### 2.3 Optimization of producing condition for HP with good CBR

The effects of different enzymes on the CBR of HP were studied. Briefly, 10 grams of *Hericium erinaceus* proteins were fully dissolved in distilled water (200 mL). Each enzyme was added to the solution (1 × 10^5^ U/g protein), and *Hericium erinaceus* proteins were hydrolyzed for 2 h. The hydrolysis condition for each enzyme was as follows: papain (50°C, pH 7.5), Protamex (55°C, pH 7), pepsin (37°C, pH 2), neutral protease (50°C, pH 7), alkaline protease (55°C, pH 8), and trypsin (37°C, pH 6.5). After inactivating enzymes (100°C, 10 min), each mixture was centrifuged (3,000 × *g*, 15 min) to collect the supernatant. After lyophilization, the CBR of the peptides in the resulting samples was analyzed, and *Hericium erinaceus* peptides released by papain (HPP) had the highest CBR, which was selected for further studies.

The effects of different hydrolysis durations on the CBR of HP were further studied. *Hericium erinaceus* proteins were hydrolyzed by papain according to the above process for 0, 0.25, 0.5, 1, 2, 3, 4, and 5 h. The CBRs of the HPP samples were compared to select the optimal hydrolysis duration.

### 2.4 Optimization of *Hericium erinaceus* peptide–calcium chelates preparation

The preparation condition of HP-Ca was optimized by single-factor and BDD experiments. Briefly, HPP was fully mixed with deionized water (10 mg/mL), followed by the addition of CaCl_2_ with the mass ratio of peptide/CaCl_2_ (1:1–6:1). The peptides reacted with CaCl_2_ at the pH of 4–9 and temperature of 30–80°C for 20–70 min. Then, absolute ethanol was added to the resulting solution in a ratio of 10:1 (v/v) to isolate the chelates. After centrifugation (9,000 × *g*, 15 min), the collected sediment was freeze-dried to prepare HP-Ca.

The single-factor experiment was conducted with one factor varying and the other three factors fixed. The CBR was used as an evaluation indicator. The basic conditions of the single-factor test were as follows: pH 8, time 40 min, temperature 50°C, and mass ratio of peptide/CaCl_2_ 2:1. After that, taking the CBR (Y, %) as the dependent variable, the condition for the reaction of HP-Ca was investigated by BBD with three independent variables: mass ratio of peptide/CaCl_2_ (A), temperature (B), and pH (C). Table 1 shows the levels of the three factors and BBD experiments.

**TABLE 1:** Results of Box–Behnken experiments.

Number	A Mass ratio of peptide/calcium	B Temperature (°C)	C pH	CBR (%)
1	2	40	8	51.68 ± 1.24
2	4	40	8	71.35 ± 1.54
3	2	60	8	51.38 ± 2.80
4	4	60	8	84.06 ± 0.69
5	2	50	7	49.46 ± 0.31
6	4	50	7	68.76 ± 1.70
7	2	50	9	47.91 ± 0.39
8	4	50	9	77.62 ± 0.76
9	3	40	7	52.77 ± 0.43
10	3	60	7	52.22 ± 0.50
11	3	40	9	53.54 ± 0.88
12	3	60	9	53.09 ± 0.51
13	3	50	8	60.55 ± 1.57
14	3	50	8	53.32 ± 0.69
15	3	50	8	56.96 ± 0.16
16	3	50	8	56.69 ± 0.98
17	3	50	8	56.32 ± 0.23

### 2.5 CBR assay

The CBR was measured following a previously reported method with slight modifications (6, 9). HP solution (0.05 g/mL, 10 mL) was mixed with CaCl_2_ solution (0.05 g/mL, 2.5 mL) and reacted for 40 min (pH8, 50°C) under stirring. Then, absolute ethanol (112.5 mL) was poured into the mixture, followed by the centrifugation (10,000 × *g*, 15 min). The concentration of Ca in the precipitate (the bound Ca) and the total Ca were measured by atomic absorption spectrophotometer:


(1) $$\mathrm{Calcium}-\mathrm{binding}\mathrm{rate}\left(\%\right)=\frac{T\,h\,e\,a\,m\,o\,u\,n\,t\,o\,f\,b\,o\,u\,n\,d\,C\,a\,\left(m\,g\right)}{T\,h\,e\,a\,m\,o\,u\,n\,t\,o\,f\,t\,o\,a\,t\,l\,C\,a\,\left(m\,g\right)}\times 100\%$$

### 2.6 Measurement of HD

Hydrolysis degree was measured as previously described (15). Briefly, *Hericium erinaceus* protein hydrolysates (0.25 mL) were mixed with 12.25 mL of sodium dodecyl sulfate solution (0.01 g/mL). Then, phosphate solution (1 mL, pH 8.2, 0.2 mol/L) and trinitrobenzene sulfonic acid (1 mL, 1 mg/g) were added to the mixture. The reaction occurred in the dark (50°C, 60 min), followed by adding HCl solution (2 mL, 0.1 mol/L). The absorbance of the final solution was measured at 420 nm. The standard solution was L-leucine with a concentration 0–5 mmol/L.

### 2.7 Measurement of amino acids

The amino acid profiles of HPP and HP-Ca were measured according to the study by Zhang et al. (8). Briefly, the two samples were hydrolyzed with HCl (6 mol/L, 10 mL) in the atmosphere of N_2_ for 24 h. The hydrolysates were passed through filter membranes (0.45 μm). Then, the contents of the amino acids were measured by an automatic amino acid analyzer (LA8080, Hitachi, Japan).

### 2.8 Structural characterization

#### 2.8.1 Morphology and EDS assay

The morphology and element distribution of HPP and HP-Ca were analyzed by a scanning electron microscope (SEM) (Gemini sigma 300, ZEISS, Germany) equipped with EDS (Xplore15, Oxford, England). Briefly, HPP and HP-Ca (5 mg) were sprayed with gold (15 mA, 90 s). The SEM images of the samples were photographed by the EDS test with an accelerating voltage of 5 kV.

#### 2.8.2 UV absorption and FT-IR assay

*Hericium erinaceus* peptides released by papain and HP-Ca were dissolved in deionized water (1 mg/mL). The UV spectra ranging from 190 to 400 nm were measured by a UV spectrophotometer (UC-5500, Yuan Instrument Co., Ltd., China) (14).

The FT-IR assay of HPP and HP-Ca was conducted as previously described (16). Briefly, HPP and HP-Ca (2 mg) were mixed with KBr (100 mg) and grounded. The samples were scanned by an FT-IR (IS5, Thermo-fisher, USA) from 4,000 cm^–1^ to 400 cm^–1^.

#### 2.8.3 XRD and thermogravimetric assay

The X-ray diffraction (XRD) data of HPP and HP-Ca were determined by an X-ray diffract meter (Bruker, Germany) under the following conditions: scan angle (2θ) (4–7°), voltage 40 kV, current 40 mA, and scanning speed 2/min (17).

The thermal property of HPP (5 mg) and HP-Ca (5 mg) was determined by a TGA (TA/Q600, TA Instruments, USA) from 25 to 600°C at the speed of 10°C/min under nitrogen (18).

### 2.9 Property and activity evaluation

#### 2.9.1 Prevention of calcium phosphate crystallization

The inhibitory effect of HPP on calcium phosphate precipitation was evaluated by a previous method (19). Briefly, HPP (20 mg) was dissolved in CaCl_2_ solution (0.8 mol/L, 2 mL), followed by adjusting the pH to 7. After incubation for 5 min (37°C), NaH_2_PO_4_ solution (0.008 mol/L, 200 mL) was added. The pH of the reaction solution was monitored for half an hour and recorded every 5 min. The distilled water was used as a blank control.

#### 2.9.2 *In vitro* digestion stability assay

The gastric digestion (GD), intestinal digestion (ID), and gastrointestinal digestion (GID) stability of HP-Ca were evaluated as previously described (20). Briefly, HP-Ca solution (0.5 mg/L) was prepared using deionized water. In the section of GD, pepsin solution (1 mg/mL) was added to the HP-Ca solution at a ratio of 1:50 (enzyme/chelates, w/w), followed by adjusting pH to 2, and the mixture was digested at 37°C for 2 h under stirring. In the section of ID, trypsin solution (1 mg/mL) was poured into the HP-Ca solution at a ratio of 1:50 (enzyme/chelates, w/w), followed by adjusting pH to 7, and the mixture was digested at 37°C for 2 h under stirring. In the section of GID, HP-Ca was sequentially digested by pepsin and trypsin following the above method. The undigested HP-Ca in each section was precipitated by the addition of nine times the volume of absolute ethanol. The retained calcium in HP-Ca was determined by an atomic absorption spectrophotometer. The digestive solution replaced by deionized water in each section was used as a blank control, and CPP-Ca was used as a positive control.


(2) $$\mathrm{The}\mathrm{calcium}\mathrm{retention}\mathrm{rate}\left(\%\right)=\frac{A\,m\,o\,u\,n\,t\,o\,f\,c\,a\,l\,c\,u\,i\,m\,i\,n\,d\,i\,g\,e\,s\,t\,e\,d\,s\,a\,m\,p\,l\,e\,s}{T\,o\,t\,a\,l\,a\,m\,o\,u\,n\,t\,o\,f\,c\,a\,l\,c\,u\,i\,m\,i\,n\,s\,a\,m\,p\,l\,e\,s}\times 100$$

#### 2.9.3 Calcium absorption assay

##### 2.9.3.1 Cell culture and viability assay

Caco-2 cells were cultured at 37°C with 5% CO_2_. The culture medium was the DMEM medium supplemented with 15% FBS, 1% NEAA, and 1% penicillin–streptomycin. Caco-2 cells grown in 96-well plates (8,000 cells/well) were cultured for 24 h. Then, the cells were treated with various amounts of HPP (0–32 mg/mL) for 24 h. The viability of the cells was evaluated using the CCK8 kit, as reported previously (21).

##### 2.9.3.2 Intracellular calcium absorption assay

The intracellular calcium absorption of HP-Ca was measured according to the method reported previously, with some alterations (19, 22). Briefly, HP-Ca was added to Caco-2 cells and incubated for 2 h. The final amounts of added calcium were 0.135–0.540 mg/mL. Then, un-absorbed HP-Ca was washed with HBSS. Fluo-3AM (5 μM, 100 μL), a Ca^2+^ binding dye, was added to the cells and treated for 1 h (37°C), followed by the supplementation of HBSS (100 μL). The cells were incubated for another 0.5 h. The fluorescence intensity of absorbed calcium in the cells was measured by flow cytometry. The cells untreated with samples were taken as a control. The fluorescence intensity of the treated groups relative to the control represents the intracellular calcium absorption. CPP-Ca and CaCl_2_ were a positive and negative control, respectively.

### 2.10 Measurement of calcium content

Briefly, HP-Ca (0.5 g) was digested by 5 mL of nitric acid in a microwave digestion instrument (MDS-15, SINEO China), following the instructions of the manufacturer. The digestion solution was further heated at 160°C until the volume was 1 mL. The calcium content of HP-Ca was measured by an atomic absorption spectrophotometer. The calcium content of the chelate was calculated following the formula:


(3) $$\mathrm{Calcium}\mathrm{content}\left(\%\right)=\frac{T\,h\,e\,w\,e\,i\,g\,h\,t\,o\,f\,c\,a\,l\,c\,i\,u\,m\,i\,n\,H\,P-C\,a\,s\,a\,m\,p\,l\,e}{T\,h\,e\,w\,e\,i\,g\,h\,t\,o\,f\,H\,P-C\,a\,s\,a\,m\,p\,l\,e}\times 100\%$$

### 2.11 Statistical analysis

All experiments were conducted in triplicate. The data were statistically evaluated by SPSS 20 (IBM Corporation, USA) at *p* < 0.05.

## 3 Results and discussions

### 3.1 Effects of different enzymes and hydrolysis duration on the CBR of HP

**FIGURE 1:**
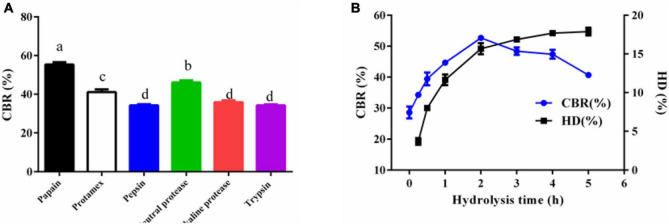
**(A)** The CBR of *Hericium erinaceus* peptides released by different proteinases (papain, Protamex, pepsin, neutral protease, alkaline protease, and trypsin) and **(B)** the effect of hydrolysis time and HD on the CBR of HPP. Different letters indicate significant differences between groups (*p* < 0.05).

Figure 1A shows the effects of different enzymes on the CBR of HP. HPP displayed the highest CBR (55.38 ± 1.21%) compared to the others (*p* < 0.05) (Figure 1A). The calcium-binding ability of the peptides was affected by enzymes, while the optimal enzyme often varied with the origin of proteins. For example, papain, neutral proteinase, and trypsin were preferred to release calcium-binding peptides from Tilapia bone collage (11), egg white (14), and cod skin gelatin (7), respectively. Considering the cost, we prefer a single enzyme to prepare calcium-binding peptides. Furthermore, Figure 1B shows that peptides hydrolyzed for 2 h with proper HD displayed the highest CBR. More hydrolysis time and higher HD means the release of more small peptides. Previous studies showed that properly hydrolyzed peptides displayed high CBR rather than the ones with higher or lower HD (6, 9). The results herein also confirmed the previous findings. Thus, papain and a hydrolysis duration of 2 h were used in the following study.

### 3.2 Single-factor experiment

**FIGURE 2:**
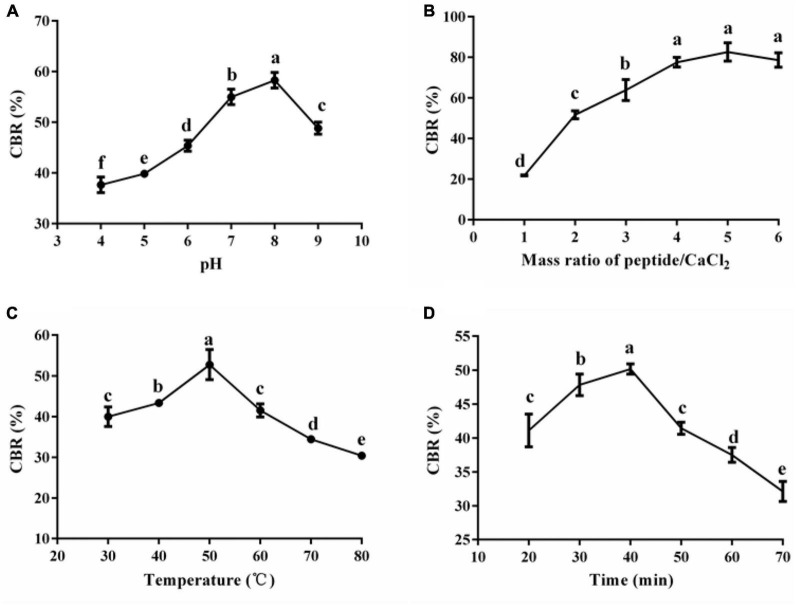
Effects of pH **(A)**, mass ratio of peptide/CaCl_2_ **(B)**, temperature **(C)**, and time **(D)** on the CBR of HPP. Different letters indicate significant differences between groups (*p* < 0.05).

Reaction conditions (temperature, pH, and mass ration of peptide/CaCl2) determine the reaction efficiency between peptides and Ca^2+^. Figure 2 shows the results of the single-factor experiment. The CBR topped at the pH of 8 (58.28 ± 1.54%) and decreased when the pH was further raised (Figure 2A). Consistently, alkaline condition (pH more than 8) facilitates the coordination reaction between Ca^2+^ and carboxyl/amino, thus improving the CBR, but strong alkaline leads to the formation of Ca(OH)_2_ rather than peptide–calcium complexes (6, 23). Figure 2B shows that the CBR dramatically increased with a mass ratio of peptide/CaCl_2_ (1:1–4:1) (*p* < 0.05) initially and there were no significant changes afterward (4:1–6:1) (*p* > 0.05). These data indicate that enough peptides were helpful for the chelation; once the amount of peptides met the needs of chelation, improvement of the ratio of peptide/CaCl_2_ had no significant effect on the increase of CBR. Similar trends were reported in peptide–calcium complexes made of pig bone collagen (6). Aiming to save the peptides, we selected the level (2:1–4:1) of the ratio of peptide/CaCl2 in the following study. The CBR varied with the increase of temperature and topped at 50°C (52.76 ± 1.77%) (Figure 2C). Proper temperature favors chelation by promoting molecular motion, while excess high temperature might make the protein conformation change, leading to a drop in chelation (24). The reaction time also affected the CBR of HPP, and the highest CBR was 50.18 ± 0.75% when the time was 40 min (Figure 2D). The chelation between calcium and peptides is a fast reaction, and a longer duration is not suitable for chelation (23). Similar results were reported by others (6); thus, 40 min was selected in the following study.

### 3.3 Response surface optimization

**FIGURE 3:**
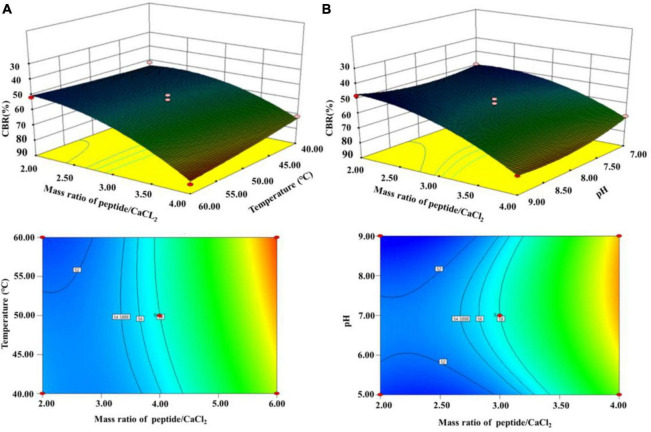
Interactive effects of **(A)** temperature and mass ratio of peptide/CaCl2 and **(B)** mass ratio of peptide/CaCl_2_ and pH on the CBR of HPP.

According to the results above, (A) a mass ratio of peptide/CaCl_2_ of 2:1–4:1, (B) temperature of 40–60°C, and (C) pH of 7–9 were chosen in BBD, and the results are shown in Table 1. The fitted model for CBR (Y) and factors were acquired: *Y* = 57.68+12.67A+1.43B+1.12C+3.25AB+2.60AC+7.30A^2^-4.41C^2^. The *p*-value (0.0002) of the model and lack of fit (0.926) were less and greater than 0.05 (Table 2), respectively, thus validating this model (21). The R^2^ (0.93) and adjusted R^2^ (0.90) were near to 1 (Table 2), confirming the good fitness of the model (21). Figure 3 shows the interactive effect between the two factors. The response surfaces were relatively flat (Figure 3), illustrating that the interactive effects were not significant (25). The speculation was also confirmed by the *p*-value of AB (0.1218) and AC (0.2049), both of which were higher than 0.05 (Table 2). Furthermore, the optimal preparation condition of HPP-Ca was acquired by the following model: mass ratio of peptide/CaCl_2_ 4:1, pH 8.5, and temperature 55°C. The CBR of HPP was 82.11 ± 1.52% under the optimized condition. The value was higher than that of grape seed peptide-Ca chelates (14.42%) (26) and egg white peptide-Ca chelates (71.93%) (14). Moreover, there was 6.79 ± 0.13% of calcium in HP-Ca, which was more than 10 times as much as HPP (0.62 ± 0.13%) (*p* < 0.05) (Table 3), confirming the successful preparation of the chelates. The calcium content in HP-Ca was also higher than egg white peptide-Ca chelates (4.41%) (14) and peptide-Ca made of sheep bone collagen (2.21%) (17). In all, these results indicate that *Hericium erinaceus* is suitable for the preparation of peptide-Ca chelates.

**TABLE 2:** Regression model and variance analysis of the CBR.

Source	Sum of squares	df	Mean square	F value	*P*-value
Model	1675.06	7	239.01	16.48	0.0002
A	1284.23	1	1284.23	88.55	<0.0001
B	16.27	1	16.27	1.12	0.3171
C	10.01	1	10.01	0.69	0.4245
AB	42.32	1	42.32	2.92	0.1218
AC	27.09	1	27.09	1.87	0.2049
A^2^	225.29	1	225.29	15.53	0.0034
C^2^	82.04	1	82.04	5.66	0.0413
Residual	130.53	9	14.50		
Lack of fit	29.91	5	5.98	0.24	0.9264
Pure error	100.62	4	25.15		
Cor total	1803.57	16			
*R*^2^ = 0.93, *R*_*adjusted*_ = 0.90, C.V. = 6.45%					

**TABLE 3:** Calcium content of HPP and HP-Ca.

Sample	Calcium content (%)
HPP	0.62 ± 0.13^b^
HP-Ca	6.79 ± 0.13^a^

Values followed by different lowercase letters mean significant differences, *p* < 0.05.

### 3.4 The content of amino acids

Peptide-Ca chelates are formed via the chelation between amino acids and Ca^2+^; thus, the profiles of amino acids were measured. Table 4 shows that the contents of Ser and the amino acids Glu and Asp in HP-Ca were significantly improved compared with HPP (*p* < 0.05), indicating that these acids were enriched after chelation. The two acidic amino acids (Glu and Asp) were involved in the chelation with Ca^2+^ because they have active carboxyl groups, which facilitate the binding of Ca^2+^ (4, 17). Many calcium-binding peptides often contain these two amino acids, for example, Asn-Asp-Glu-Glu-Leu-Asn-Lys and Ser-Ser-Ser-Asp-Asp (11, 23). Moreover, previous studies reported that the CBR of soybean peptides was positively related to the proportion of Glu and Asp (27). Ser is another amino acid commonly found in peptides with metal-binding ability, and both the amount and location of phosphoserine residues are directly related to this capability of peptides (28, 29). Thus, the result herein further confirms the successful chelation between Ca^2+^ and HPP and illustrates that Ser and the two acidic amino acids Glu and Asp might be involved in the chelation with Ca^2+^.

**TABLE 4:** Amino acids content of HPP and HP-Ca.

Amino acids	HPP (%, w/w)	HP-Ca (%, w/w)
Glu	8.01 ± 0.13^a^	10.78 ± 0.24^b^
Asp	9.29 ± 0.08^a^	11.64 ± 0.17^b^
Ser	4.51 ± 0.10^a^	5.56 ± 0.06^b^
Gly	3.45 ± 0.02^a^	3.61 ± 0.05^a^
Thr	4.56 ± 0.11^a^	4.4 ± 0.09^a^
Ala	5.75 ± 0.16^a^	5.2 ± 0.30^a^
Arg	7.15 ± 0.21^a^	5.5 ± 0.32^b^
Pro	4.88 ± 0.33^a^	2.66 ± 0.45^b^
Cys	0.03 ± 0.001^a^	0.04 ± 0.001^a^
Lys	4.72 ± 0.34^a^	4.97 ± 0.60^a^
Met	–	0.01 ± 0.001
Val	5.53 ± 0.26^a^	5.52 ± 0.19^a^
Tyr	3.04 ± 0.06^a^	2.64 ± 0.44^a^
Ile	4.19 ± 0.31^a^	2.51 ± 0.08^b^
Leu	7.39 ± 0.22^a^	5.48 ± 0.41^b^
Phe	4.63 ± 0.10^a^	2.79 ± 0.06^b^
His	1.88 ± 0.04^a^	3.03 ± 0.07^b^

Values followed by different lowercase letters mean significant differences, *p* < 0.05; “–” means not detected.

### 3.5 Characterization of HP-Ca

#### 3.5.1 Morphology and EDS assay

**FIGURE 4:**
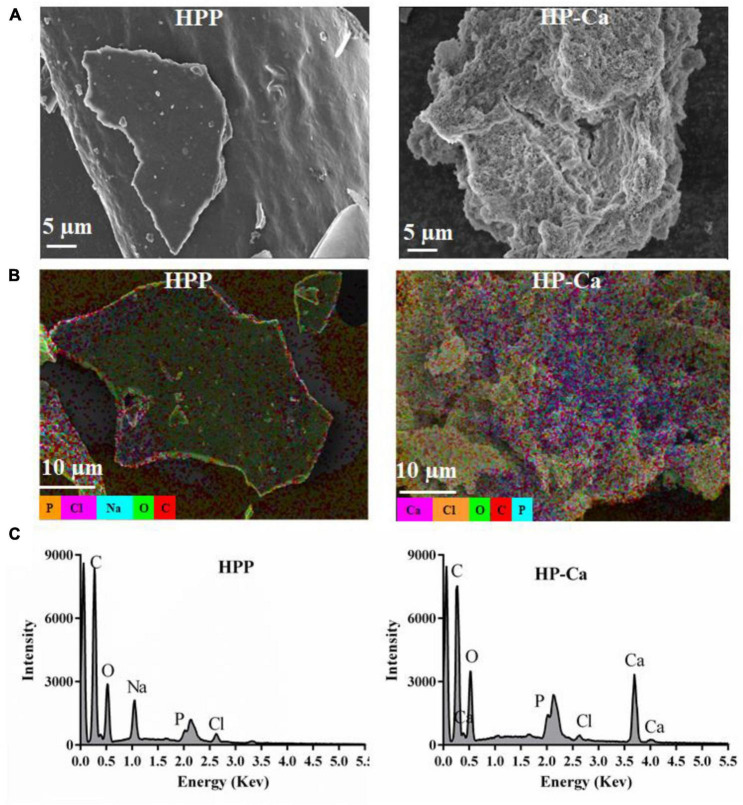
Morphology and EDS analysis of HPP and HP-Ca. **(A)** SEM images of HPP and HP-Ca. **(B)** EDS layered images of HPP and HP-Ca. **(C)** Elements signal intensity in the EDS image of HPP and HP-Ca.

The morphology of HPP and HP-Ca was examined by SEM. Figure 4A shows that HPP was in a compact plate shape with a smooth surface. However, the microstructure of HP-Ca was loose and porous (Figure 4A). The dramatic changes might be due to the interaction of Ca with HPP, leading to the destruction of the original microstructure (28). Similar morphological changes have also been reported when herring egg phosphopeptides chelate with calcium (28). Furthermore, EDS was used to analyze the element distribution of HPP and HP-Ca. Figure 4B clearly shows that there was plenty of calcium (colored purple) distributed in HP-Ca, but no calcium existed in HPP. Moreover, the signal of calcium was observed in HP-Ca but not in HPP (Figure 4C). These data confirm the successful chelation of calcium with HPP.

#### 3.5.2 UV absorption and FT-IR assay

**FIGURE 5:**
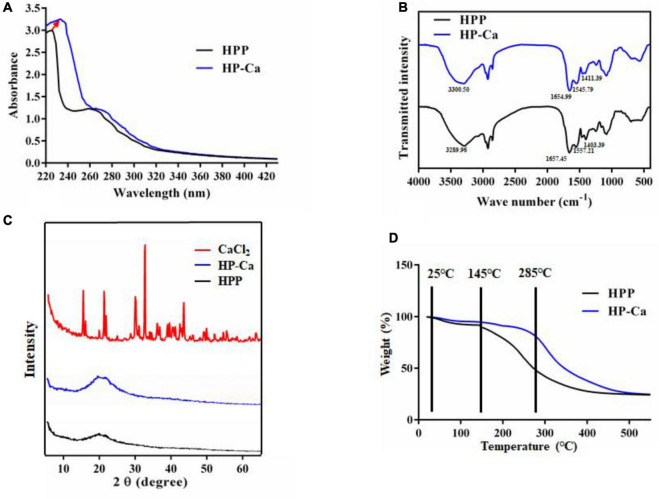
Characterization of HPP and HP-Ca. **(A)** UV scanning analysis of HPP and HP-Ca. **(B)** FT-IR spectroscopy of HPP and HP-Ca. **(C)** XRD analysis of HPP and HP-Ca. **(D)** Thermogravimetric analysis of HPP and HP-Ca.

The possible functional groups involved in chelation were further studied by UV and FT-IR assay. As shown in Figure 5A, a strong absorption at 224 nm was found in HPP, which resulted from the n→π* transition of carboxyl and *C* = O in the amide bond (9). Another weak absorption was found at 260 nm rather than at 280 nm for HPP (Figure 5A), which might be attributed to the higher amount of Phe than Tyr in HPP (4.63 ± 0.10% *vs.* 3.04 ± 0.06%) (Table 4). In other studies, wheat germ protein hydrolysates have been found to also have their maximum absorption at 260 nm (9). After reacting with calcium, the maximum absorption shifted to 233 nm, and the absorption intensity improved (Figure 5A), further confirming the chelation between Ca^2+^ and HPP. These might be due to the polarizing changes of carboxyl and the amide bond induced by Ca^2+^ after chelation (18, 19). Moreover, the original weak absorption peak at 260 nm shifted near to 280 nm (Figure 5A), which might be due to the increase of the ratio of Tyr to Phe (2.79 ± 0.06% *vs.* 2.64 ± 0.44%) in HP-Ca (Table 4). Collectively, carboxyl and *C* = O in the amide bond of HPP might be involved in chelation with Ca^2+^.

Fourier transform infrared spectroscopy is often used to reveal the binding sites or bonds between peptides and calcium (30). Figure 5B shows the FT-IR profiles of HPP and HP-Ca. After chelating with calcium, amide band I at 1,657 cm^–1^ resulting from C = O shifted to 1,654 cm^–1^, and amide band II attributed to the bending vibration of N-H shifted from 1,557 cm^–1^ to 1,545 cm^–1^ (Figure 5B). Thus, the C = O and N-H might be involved in the reaction and provide chelating sites for calcium, respectively. The absorption at 1,403 cm^–1^ was caused by the vibration of –COO^–^ (30, 31). Once reacted with Ca^2+^, it changed to 1,411 cm^–1^, indicating that –COO^–^ might also interact with calcium by ionic bond or electrostatic effect. Because free -COO^–^ is usually provided by acidic acids, these data further confirm that Glu and Asp were involved in the chelation. Collectively, these analyses confirm the successful preparation of HP-Ca chelates and that Ca^2+^ binds to HPP via interacting with free -COO^–^ from acidic amino acids and C = O from amide.

#### 3.5.3 XRD and thermogravimetric assay

X-ray diffraction was applied in confirming the production of new substance by monitoring the changes of the X-ray spectra (16). CaCl_2_ displayed many sharp and strong diffraction peaks, while these sharp peaks disappeared in the spectra of HP-Ca, and only a gentle and weak band was found in the of HP-Ca and HPP (Figure 5C). These data indicate that CaCl_2_ was not mixed physically with HPP but formed new compounds via chelation, and the chelated calcium was in amorphous form. Others also found the disappearance of crystal structure when CaCl_2_ chelated with glycosylated peptide (19).

The thermal stability of HPP and HP-Ca was evaluated. The weight of HPP decreased slowly from 25 to 145°C, and the sharp weight loss happened afterward (Figure 5D). However, the sharp weight loss of HP-Ca began at a higher temperature (285°C), indicating that chelation with calcium may enhance the thermal stability of HPP (Figure 5D). These might be due to the formation of new bonds between HPP and Ca^2+^, and disruption of the chelates requires more energy (30). Similarly, peptide-Ca chelates made of cattle bone collagen was found to show higher stability than collagen peptides (30). Moreover, thermal stability of Gly-Try was also improved when chelated with Ca^2+^ (10). These previous studies are consistent with our results.

### 3.6 HPP prevents calcium phosphate crystallization

**FIGURE 6:**
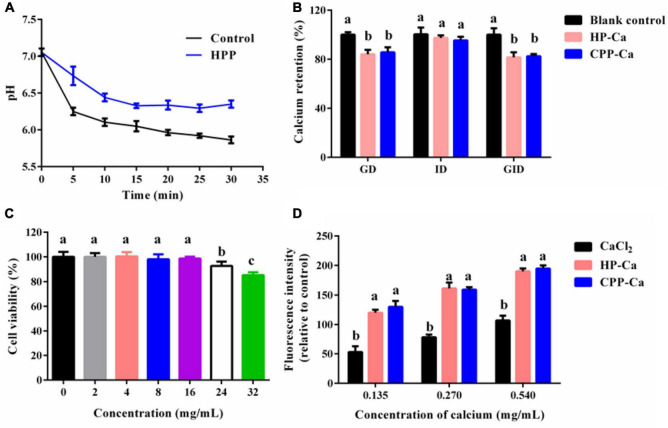
**(A)** Inhibitory effect of HPP on calcium phosphate crystallization. **(B)** Stability of HP-Ca against simulated gastrointestinal digestion. **(C)** Cell viability of Caco-2 treated with various concentrations (0–32 mg/mL) of HP-Ca. **(D)** Intracellular calcium absorption of cells treated with HP-Ca, CaCl_2_, and CPP-Ca, respectively. The final amount of added calcium was 0.135, 0.270, and 0.540 mg/mL. Different letters indicate significant differences between groups (*p* < 0.05).

Ca^2+^ usually reacts with phosphoric or oxalic acid in *vivo* and produces undissolved compounds, decreasing the biological value of the metal (32). When Ca is precipitated by a phosphate group, the pH of the reaction system will decrease (33). Therefore, pH monitoring can be applied in evaluating the formation of calcium phosphate crystals (33). Figure 6A shows that pH of the reaction system dropped rapidly in the control group, indicating that Ca^2+^ can be easily precipitated by phosphate. Compared with the control, the pH of the HPP group reduced more slowly, indicating that HPP could effectively inhibit the formation of Ca_3_(PO_4_)_2_ precipitate. Thus, HPP can avoid Ca^2+^ loss in the intestine. Others also reported similar activities, such as glycosylated peptides made of crimson snapper scales (19).

### 3.7 *In vitro* digestion stability of HP-Ca

Once ingested, the acid environment and protein enzymes in the gastro-intestine will induce the liberation of Ca^2+^ from H-Ca. The released Ca^2+^ might form undissolved compounds, leading to the low bioavailability of Ca. Thus, the GD, ID, and GID stability of HP-Ca were further evaluated. Figure 6B shows that only a tiny amount of calcium (less than 18%) was lost during the GID, which was mainly due to the calcium release in the stage of GD (nearly 16%). HP-Ca was stable in the stage of ID, in which only 4% of calcium was lost (Figure 6B). Moreover, there was no difference in the calcium retention rate between HP-Ca and the positive control (CPP-Ca) in each digestion process (*p* < 0.05). These data confirm the good digestion stability of HP-Ca. Low pH (2) in GD is adverse to the CBR of HPP (Figure 2A), and the hydrolyzing of HPP by pepsin might disrupt the binding of Ca^2+^ to the chelate. These are responsible for calcium loss of HP-Ca in this stage. In addition, the alkaline condition at the ID stage is helpful for the chelation between HPP and Ca^2+^, thus maintaining the high calcium retention rate (23). Similarly, glycosylated peptide–calcium and phosvitin peptide–calcium have been shown to display good stability against stimulated GID (8, 19). Therefore, HP-Ca has good stability during GID, which is helpful for the bioavailability of Ca.

### 3.8 Intracellular calcium absorption of HP-Ca

Furthermore, Caco-2 cells were used for the measurement of the intracellular calcium absorption of HP-Ca. Aiming to avoid the possible influence of HP on cells, the optimal concentration of HP was analyzed. As shown in Figure 6C, 2–16 mg/mL of HP had no significant effects on cell viability (*p* < 0.05); thus, concentrations of 2–8 mg/mL were used. Figure 6D shows that, compared with ionic calcium (CaCl_2_), the fluorescence intensity of absorbed calcium in both the HP-Ca and CPP-Ca groups was improved significantly (*p* < 0.05) in a dose-dependent manner. CPP-Ca is a good calcium supplement and has been sold in the market. There was no significant difference between the two chelated Ca groups (*p* > 0.05), indicating that HP-Ca could effectively facilitate the uptake of calcium. Similar trends were found in the study of peptide-Ca made of bone collagen (11) and crimson snapper scales (20). Peptides can be absorbed directly by the intestine via endocytosis, and calcium-binding peptides could serve as calcium carriers to improve calcium absorption in the intestine (20, 22). Generally, Ca^2+^ is mainly absorbed by enterocytes via calcium channels *in vivo*, such as TRPV6 and Cav1.3. Peptide-Ca chelates or calcium-binding peptides can interact with calcium channels to improve Ca absorption (4). For example, the sub-fraction of CPP (a commercial calcium-binding peptide) improved Ca absorption by regulating TRPV6 expression (20, 34). In addition to regulating calcium channels, peptides can also serve as activators of calcium-sensing receptors to promote Ca uptake (35). Nonetheless, the underlying mechanisms for the improvement of Ca uptake by HPP-Ca need to be further investigated.

## 4 Conclusion

In this study, the preparing condition, structure, and physical properties of HP-Ca were investigated for the first time. It was found that calcium possibly chelated to HP via interaction with free -COO- from acidic amino acids and C = O from amide. HP-Ca was more resistant to heat and had a good gastrointestinal stability *in vitro*. Moreover, HP-Ca could effectively facilitate the absorption of calcium. Thus, HP-Ca is a promising calcium supplement with high bioavailability. The study provides a new idea to develop peptide-Ca supplements by edible fungus. Meanwhile, the possible mechanism for high Ca absorption of the chelate should be investigated in the future.

## Data availability statement

The original contributions presented in this study are included in this article/supplementary material, further inquiries can be directed to the corresponding authors.

## Ethics statement

Ethical approval was not required for the studies on animals in accordance with the local legislation and institutional requirements because only commercially available established cell lines were used.

## Author contributions

HG: Conceptualization, Funding acquisition, Supervision, Writing—original draft, Writing—review and editing. LL: Conceptualization, Data curation, Funding acquisition, Writing—review and editing. YK: Formal analysis, Investigation, Methodology, Writing—original draft. RY: Data curation, Formal analysis, Investigation, Methodology, Writing—review and editing. JW: Investigation, Methodology, Writing—review and editing. DF: Formal analysis, Investigation, Methodology, Writing—review and editing.
